# Customized Steady-State Constraints for Parameter Estimation in Non-Linear Ordinary Differential Equation Models

**DOI:** 10.3389/fcell.2016.00041

**Published:** 2016-05-11

**Authors:** Marcus Rosenblatt, Jens Timmer, Daniel Kaschek

**Affiliations:** ^1^Institute of Physics, Albert Ludwig University of FreiburgFreiburg, Germany; ^2^Freiburg Centre for Systems Biology, Albert Ludwig University of FreiburgFreiburg, Germany; ^3^BIOSS Centre for Biological Signaling Studies, Albert Ludwig University of FreiburgFreiburg, Germany

**Keywords:** non-linear ODE models, parameter estimation, biochemical reaction networks, steady-state, positive solutions, multiplicity, multi-stability, success rate

## Abstract

Ordinary differential equation models have become a wide-spread approach to analyze dynamical systems and understand underlying mechanisms. Model parameters are often unknown and have to be estimated from experimental data, e.g., by maximum-likelihood estimation. In particular, models of biological systems contain a large number of parameters. To reduce the dimensionality of the parameter space, steady-state information is incorporated in the parameter estimation process. For non-linear models, analytical steady-state calculation typically leads to higher-order polynomial equations for which no closed-form solutions can be obtained. This can be circumvented by solving the steady-state equations for kinetic parameters, which results in a linear equation system with comparatively simple solutions. At the same time multiplicity of steady-state solutions is avoided, which otherwise is problematic for optimization. When solved for kinetic parameters, however, steady-state constraints tend to become negative for particular model specifications, thus, generating new types of optimization problems. Here, we present an algorithm based on graph theory that derives non-negative, analytical steady-state expressions by stepwise removal of cyclic dependencies between dynamical variables. The algorithm avoids multiple steady-state solutions by construction. We show that our method is applicable to most common classes of biochemical reaction networks containing inhibition terms, mass-action and Hill-type kinetic equations. Comparing the performance of parameter estimation for different analytical and numerical methods of incorporating steady-state information, we show that our approach is especially well-tailored to guarantee a high success rate of optimization.

## 1. Introduction

Dynamical systems are frequently modeled by systems of ordinary differential equations (ODEs). Homogeneously distributed molecules are treated as continuous quantities interacting with each other according to kinetic laws, e.g., mass-action or Michaelis-Menten kinetics.

A typical ODE system

(1) $$\begin{array}{ll} ẋ=f\left(x,p,u\left(t\right)\right),\;x\left(0\right)=x_{0} & \; \end{array}$$

determines the time-evolution of an *N*-dimensional state vector *x*(*t*). Here, $p\in {\mathbb{R}}_{+}^{M}$ denotes the *M*-dimensional vector of non-negative kinetic parameters. The vector $x_{0}\in {\mathbb{R}}_{+,0}^{N}$, where ℝ_+, 0_ = ℝ_+_ ∪ {0}, gives the set of initial conditions. The kinetic parameters and initial conditions together span the space of model parameters θ = (*p, x*_0_). The explicit time-dependency via *u*(*t*) corresponds to external driving forces, like drug stimuli in biological dynamic systems.

In many fields where ODE models are used, parameter values are not *a priori* known and have to be estimated from experimental data. Commonly, this is achieved by minimizing an objective function *g*(θ, *D*) that penalizes weighted differences between model prediction *x*(*t*) and data *D*, e.g., by maximum-likelihood estimation. For the case of non-linear ODE systems, several local optima may exist. In order to find the global optimum, several optimization methods, e.g., particle swarm optimizers (Peng et al., 2010) or simulated annealing (Xiang and Gong, 2000), include stochasticity to escape local minima. Compared to that, deterministic algorithms may stick to local optima during optimization. On the other hand, gradient and Hessian information of the objective function can be incorporated, increasing the performance of optimization by a multiple. Combining the advantages of derivative-based optimization and random sampling, a multi-start deterministic optimization approach has proven to yield superior overall performance for our problem class (Raue et al., 2013). Throughout this work, we perform optimization by means of a trust-region optimizer from multiple starting positions.

Specially in models of biological systems, available data is sparse and parameters are often non-identifiable. Apart from that, the high-dimensional parameter space hampers parameter sampling. In order to reduce the number of parameters, the system is assumed to initially (*t* = 0) be in a steady-state which is determined by the constraint equation

(2) $$\begin{array}{ll} f\left(x_{0},p,0\right)=0. & \; \end{array}$$

As a standard approach, the steady-state constraint is solved for the initial values *x*_0_. Since Equation (2) is in general non-linear, this may lead to higher-order polynomial equations for which no general solution is available. Even for a rather simple case of quadratic or cubic equations, solutions are not unique and optimization would have to be performed for all possibilities. Another aspect of steady-state calculation are negative solutions for *x*_0_ and *p* that appear for certain model specifications. Negative solutions are not only contradicting the biological setting with positively defined concentrations and kinetic parameters but also constitute a problem for optimization. Negative parameter values change the sign of damping terms of the ODE's right-hand side which might lead to rapidly growing solutions and an abort of the optimization before an optimum was reached.

In order to obtain a high convergence probability for the optimization of randomly chosen initial parameter samples, our aim is to derive non-negative, analytical steady-state expressions, while multiple steady-state expressions are likewise circumvented by a proper choice of kinetic and initial value parameters for which Equation (2) is solved.

Over the last decades, steady-state analysis has been addressed by many algorithms and methods. In the following, we give an overview of existing approaches and summarize their applicability to different types of model equations with a special focus on parameter estimation in ODE models, see Table 1.

**Table 1:** **Covered reaction types and positivity for different methods of steady-state determination**.

**Method**	**CRN, Mass action, Production, Degradation**	**Inhibition, Michaelis-Menten**	**Hill Kinetic**	**Non-negative Solutions**
King-Altman	Yes	No	No	Yes
Feliu and Wiuf	Yes	No	No	Yes
Halasz et al.	Yes	Yes	No	No
Loriaux et al. (*py-substitution*)	Yes	Yes	Yes	No
Proposed	Yes	Yes	Yes	Yes

The earliest-proposed algorithm for deriving steady-states in enzyme-catalyzed systems being described by simple mass-action rules was developed by King and Altman (1956). In the original paper, however, interactions that do not involve the enzyme were not allowed which prohibits applicability to most of today's systems with proteins mediating the activation of other proteins without being part of the reaction. After Chemical Reaction Network Theory (CRNT) was formulated (Horn and Jackson, 1972; Feinberg, 1979), the method of King and Altman has been improved by graph theory (Chou, 1990) and extended to special subclasses of CRNs, e.g., layered signaling cascades (Feliu et al., 2012) and post-translational modification networks (Feliu and Wiuf, 2013). The same authors also published a more general approach for CRNs in Feliu and Wiuf (2012). Here, a set of core variables is introduced serving for a parametrization of the steady-states whereby non-negative solutions are guaranteed due to graph-theoretical arguments.

Another approach developed by Halasz et al. (2013), introduces bilinearities of the system as new variables leading to a linearized system solvable by application of Cramer's rule. The number of bilinearities, however, is restricted and negative steady-state solutions are not prevented.

All mentioned approaches deal with steady-state analysis for CRNs based on mass-action rules. However, modern modeling approaches often make use of special reaction types such as inhibition, Michaelis-Menten or Hill kinetics that cannot be included into standard CRNT without changing the model structure and introducing new dynamical variables. In the approach of Halasz et al. (2013), inhibition and Michaelis-Menten terms can easily be integrated by multiplying the corresponding steady-state equation by the denominator of the rate expression. However, since a state variable is contained in the denominator, this can increase the number of bilinearities significantly.

In order to avoid problems of higher-order polynomial equations, steady-state equations can be solved not only for initial value- but also for kinetic parameters, which is done in the steady-state solver *py-substitution* developed by Loriaux et al. (2013). From *N* initial values and *M* kinetic parameters, a set of *N* variables is chosen that have to be fixed in Equation (2). In doing so, a lot of freedom is incorporated into the solution. In fact, *py-substitution* is able to solve the very most steady-state equation systems, since in principle *N* kinetic parameters could be chosen as fixed variables directly leading to a simple linear equation system.

Complementary to analytical approaches, steady-state information can be incorporated into the system by numerically computing the initial conditions during each optimization step. Even gradient information that is necessary for efficient optimization is available by means of the implicit function theorem. A numerical incorporation of steady-state information has the advantage that the complexity of the underlying equation system is in principal not restricted. Furthermore, the implementation remains untouched when model equations are changed. However, convergence of the numerical steady-state calculation is not guaranteed and issues of multiple steady-states cannot be controlled.

In the following section, we present a method to derive non-negative steady-state expressions for a large class of nonlinear ODE models that are based on biochemical reactions. Our approach picks up the idea of solving for kinetic parameters in order to derive unique and simple steady-state expressions. Due to the structure of the ODE system, solving for kinetic parameters often leads to potentially negative steady-state solutions, depending on the point of evaluation in parameter space. By introducing appropriate parameter transformations and exploiting the given model structure, our approach guarantees a non-negative solution space. In the Results section, we show how different steady-state parameterizations influence the optimization procedure and compare our approach to the standard approach of solving for initial value parameters as well as to a numerical steady-state approach.

## 2. Methods

### 2.1. Theoretical background

Let us consider a *model f* as an *N*-dimensional ODE system *ẋ* = *f*(*x, p*) with states *x*, parameters *p* and no external driving forces, i.e., the ODE is autonomous. We write *f* as a matrix product

(3) $$\begin{array}{ll} f\left(x,p\right)=S\cdot F\left(x,p\right), & \; \end{array}$$

of the *N* × *M*-dimensional *stoichiometry matrix S* and the *M*-dimensional *flux vector F* which depends on states and parameters. For the entries of the flux vector, we allow rational functions of *x* and *p* including e.g., mass-action, inhibition, Michaelis-Menten and Hill-Type kinetics. Table 2 gives an overview of the main reaction types covered by the presented steady-state approach.

**Table 2:** **Examples of typical reaction types of ODE models**.

**#**	**Stoichiometry**	**Reaction type**	**Flux**	**Contribution to $\frac{dA}{dt}$**
1	∅ → *A*	Production	*k*	Positive/influx
2	*A* → ∅, *B*	Degradation, transformation	*k* · *A*	Negative/outflux
3	*A* + *A* → *AA*	Dimerization	*k* · *A*^2^	Negative/outflux
4	*B* → *A*	Transformation	*k* · *B*	Positive/influx
5	*B* + *C* → *A*	Binding	*k* · *B* · *C*	Positive/influx
6	*B* → *A*	Inhibition by C	$k\cdot B\cdot \frac{1}{q+C}$	Positive/influx
7	*B* → *A*	Michaelis-Menten	$k\cdot B\cdot \frac{1}{q+B}$	Positive/influx
8	*A* + *B* → *C*	Binding	*k* · *A* · *B*	Negative/outflux
9	*A* → *B*	Inhibition by C	$k\cdot A\cdot \frac{1}{q+C}$	Negative/outflux
10	*A* → *B*	Michaelis-Menten	$k\cdot A\cdot \frac{1}{q+A}$	Negative/outflux
11	*B* → *A*	Hill	$k\cdot \frac{B^{q_{2}}}{{q_{1}}^{q_{2}}+B^{q_{2}}}$	Positive/influx
12	∅ → *A*	Self-activation	$k\cdot \frac{A^{q_{2}}}{{q_{1}}^{q_{2}}+A^{q_{2}}}$	Positive/influx
13	*B* + *C* → *A*	Power-law	$k\cdot B^{q_{1}}\cdot C^{q_{2}}$	Positive/influx

All types are covered by our steady-state approach.

We assume that each single flux *F*_*l*_ is proportional to some *flux parameter k*_*l*_ and can be written as

(4) $$\begin{array}{ll} F_{l}=k_{l}\cdot G_{l}\left(x,q\right), & \; \end{array}$$

where the function *G*_*l*_ only depends on the states and a set of *additional parameters q* taken from the set of all model parameters *p*. The union of flux parameters *k* and additional model parameters *q* coincides with the parameter set *p*. Typically all reaction types described by CRNT only need one flux parameter and do not contribute to *q*, however, inhibition terms and Michaelis-Menten kinetics contain at least one additional parameter and Hill kinetics even two.

The signs of the entries of the stoichiometry matrix *S* determine whether a flux contributes as an *in*- or an *outflux* to the time evolution of the corresponding state. We assume that each outflux is at least linearly dependent on the corresponding state, as being always the case for mass-action systems. By means of Equation (2), each initial value *x*_0, *i*_ ≡ *x*_*i*_ is directly related with a steady-state equation of the form

(5) $$\begin{array}{ll} 0=\sum {\text{in}}_{i}-x_{i}\cdot \sum {\text{out}}_{i}, & \; \end{array}$$

where in_*i*_ and out_*i*_ constitute functions of states and parameters. For a majority of reaction types used in ODE models, the fluxes in_*i*_ and out_*i*_ are independent of *x*_*i*_, compare Table 2. In these cases, Equation (5) is linear in *x*_*i*_ and has the solution $x_{i}=\frac{\sum {\text{in}}_{i}}{\sum {\text{out}}_{i}}$. However, if the fluxes in_i_ or out_*i*_ still depend on *x*_*i*_, e.g., reaction 12 in Table 2 for the case of self-activation or reaction 3 with an outflux being quadratic in *x*_*i*_, Equation (5) might be non-linear in *x*_*i*_.

In order to solve the complete set of steady-state equations, we analyze their specific structure by means of graph theory. We therefore rewrite Equation (5) as

(6) $$\begin{array}{ll} x_{i}=\frac{\sum {\text{in}}_{i}}{\sum {\text{out}}_{i}} & \; \end{array}$$

and summarize appearances of states on the right-hand side of Equation (6). Here, the set of states is defined by the set of dynamic variables *x* that we want to fix by the steady-state determination. Once a dynamic variable is fixed by a non-negative expression or treated as a free parameter, it is removed from the set of states.

**Definition 1:** A *head* of state *x*_*i*_ is a state *x*_*j*_ that appears on the right-hand side of Equation (6). By *h*(*x*_*i*_), we refer to the set of heads for a specific state *x*_*i*_. In particular, *x*_*i*_ can itself be a part of *h*(*x*_*i*_).

**Proposition 1:** If non-negative steady-state solutions for all heads of *x*_*i*_ are known, a non-negative steady-state solution for *x*_*i*_ can directly be obtained by Equation (6). This holds especially, if the set *h*(*x*_*i*_) is empty.

**Definition 2:** The *adjacency matrix M*(*f*) of an ODE model *f*(*x, p*) with states *x* and parameters *p* is an *N* × *N* matrix with entries

$$M_{ji}=\{\begin{array}{l} 1,\text{ if }x_{j}\in h(x_{i})\\ 0,\text{ else}. \end{array}$$

Each *d*_*M*_-dimensional adjacency matrix *M* defines a *directed graph G*_*M*_ with nodes *x*_1_ to *x*_*d*_*M*__ which we call *steady-state graph*. Each non-zero entry of *M* corresponds to a directed edge(*x*_*j*_, *x*_*i*_) implying that *x*_*j*_ occurs in the steady-state expression of *x*_*i*_, i.e., Equation (6). A non-zero diagonal entry *M*_*ii*_ reflects that the corresponding steady-state equation is non-linear in *x*_*i*_.

### 2.2. Splitting cycles

The specific structure of the steady-state graph enables to solve the steady-state equations step-by-step as is shown in the following.

**Definition 3:** A *cycle* of a steady-state graph is a path through the graph along its edges with equal starting and end point. Here, we allow cycles of length one arising from non-zero diagonal entries in the adjacency matrix *M*.

**Definition 4:** Graphs that do not contain cycles are called *tree-like*.

**Proposition 2:** If a steady-state graph of an *N*-dimensional model *f* is tree-like, non-negative steady-state solutions can be obtained for all *x*_*i*_ inside the graph.

*Proof:* For any tree-like steady-state graph, there exists at least one *root*, i.e., state without head, called *x*_*r*_. Since *h*(*x*_*r*_) = ∅, the corresponding steady-state expression can be obtained by *Proposition 1*. In doing so, *x*_*r*_ is removed from the steady-state graph and a new state serves as root for which *Proposition 1* again gives the corresponding steady-state expression. By iteratively applying *Proposition 1* for each of the *N* nodes, the complete steady-state solution is obtained.

Considering *Proposition 2*, it is clear that solving the steady-state constraint Equation (2) for the set of initial values only becomes intricate, if there are cycles inside the steady-state graph such that higher-order polynomial equations arise. The idea of our steady-state approach is to split all these cycles step-by-step such that *Proposition 2* can ultimately be applied to the remaining graph.

The simplest way of splitting a cycle is by means of a conserved quantity (CQ) of the system arising from the stoichiometry. A general introduction can be found in Loriaux et al. (2013) or Halasz et al. (2013). The following definition restricts to the properties being relevant for the presented approach.

**Definition 5:** A *conserved quantity* (CQ) of the model *f* is an expression of states and parameters which remains constant during the time-evolution of *f*. For each CQ, the number of independent steady-state equations is reduced by one implying that one state or flux parameter that appears in the CQ can be chosen freely. If all CQs can be derived from the stoichiometry matrix, the number of CQs is given by *n*_*cq*_ = *N* − *R*_*S*_, with the model size *N* and the rank of the stoichiometry matrix *R*_*S*_. The cases for which *n*_*cq*_ > *N* − *R*_*S*_ are discussed in Section 2.5.

In order to split a cycle by a CQ, one of the states, *x*_*c*_, that appears both inside the cycle and in the CQ is chosen freely. The corresponding steady-state equation is removed from Equation (2), whereby the number of independent steady-state equations remains constant. Since the state *x*_*c*_ is treated as a free variable, all edges originating from and leading to *x*_*c*_ can be removed from the steady-state graph and the considered cycle is split. Note, that each CQ can only be used once.

If no states inside the cycle appear in CQs, the cycle can be split by solving the steady-state equation of a specific cycle state *x*_*i*_ for a flux parameter *k*_*l*_. By means of Equations (3) and (4), the steady-state expression of *k*_*l*_ holds

(7) $$\begin{array}{ll} k_{l}=\frac{-1}{S_{il}G_{l}\left(x,q\right)}\underset{j\neq l}{\sum }S_{ij}k_{j}G_{j}\left(x,q\right). & \; \end{array}$$

**Proposition 3:** Let *n*_*k*_*l*__ be the number of appearances of the flux parameter *k*_*l*_, see Equation (4), inside the steady-state constraint Equation (2). Then *n*_*k*_*l*__ coincides with the number of non-zero entries in the *l*-th column of the stoichiometry matrix *S*.

Unless *k*_*l*_ does not appear in other steady-state equations, i.e., *n*_*k*_*l*__ = 1, the considered cycle is removed from the steady-state graph without affecting other parts of the graph. However, if *n*_*k*_*l*__ > 1, all further appearances have to be substituted by Equation (7) which creates new edges inside the steady-state graph and possibly even new cycles. In order to keep the structure as simple as possible, flux parameters with *n*_*k*_*l*__ = 1 play a special role.

### 2.3. Enforcing positivity

Although solving for flux parameters implies linear equations and therefore structurally simple steady-state expressions, the solutions are often negative for certain model specifications. Here, we show how positivity of the expressions can be guaranteed by appropriate transformations.

The steady-state expression, Equation (7), of the flux parameter *k*_*l*_ was derived by means of the steady-state equation of *x*_*i*_. The expression contains minus signs if and only if at least one of the stoichiometry entries *S*_*i, j*≠*l*_ has the same sign as *S*_*il*_, In this case, at least one further flux contributes to *x*_*i*_ with the same sign as *F*_*l*_ = *k*_*l*_*G*_*l*_, namely as an in- or outflux.

**Definition 6:** For the steady-state equation of *x*_*i*_, Equation (5), we define μ_*i*_ and ν_*i*_ as the number of in- and outfluxes, respectively. Furthermore, we define the *dimension* of the state *x*_*i*_ as the minimum dim(*x*_*i*_) = min(μ_*i*_, ν_*i*_).

If dim(*x*_*i*_) = 1, a non-negative steady-state expression is obtained by solving for the particular flux parameter being the only in- or outflux, compare first examples in Table 3.

**Table 3:** **Examples of solving steady-state equations for flux parameters**.

**Steady-state equation**	**Solve for**	**Type**	**Solution**
$0=k_{1}A^{2}-k_{2}X$	*k*_1_	1	$k_{1}=\frac{k_{2}X}{A^{2}}$
$0=k_{1}A^{2}-k_{2}X$	*k*_2_	1	$k_{2}=\frac{k_{1}A^{2}}{X}$
$0=k_{1}A^{2}-k_{2}X-k_{3}XB-\frac{k_{4}X}{q_{1}+C}$	*k*_1_	1	$k_{1}=\frac{X}{A^{2}}\left(k_{2}+k_{3}B+\frac{k_{4}}{q_{1}+C}\right)$
$0=k_{1}A^{2}+k_{5}B-k_{2}X\;-k_{3}XB-\frac{k_{4}X}{q_{1}+C}$	*k*_1_, *k*_5_	2	$k_{1}=\frac{1}{A^{2}}X\left(k_{2}+k_{3}B+\frac{k_{4}}{q_{1}+C}\right)\cdot \frac{1}{1+r}$
			$k_{5}=\frac{1}{B}X\left(k_{2}+k_{3}B+\frac{k_{4}}{q_{1}+C}\right)\cdot \frac{r}{1+r}$

If dim(*x*_*i*_) > 1, positivity can be enforced by performing an appropriate parameter transformation. In order to do so, we divide the fluxes contributing to *x*_*i*_ into influxes *F*_in, 1_ … *F*_in,μ_*i*__ and outfluxes *F*_out, 1_ … *F*_out,ν_*i*__. Then, Equation (5) reads

(8) $$\begin{array}{ll} 0=\overset{\mu_{i}}{\underset{j=1}{\sum }}F_{\text{in},j}-\overset{\nu_{i}}{\underset{l=1}{\sum }}F_{\text{out},l}. & \; \end{array}$$

Let us assume that we want to solve Equation (8) for the influx parameter $k_{\text{in},1}=\frac{F_{\text{in},1}}{G_{\text{in},1}}$. We perform a variable transformation by defining the ratio between the remaining influxes and *F*_in, 1_ as

(9) $$\begin{array}{ll} r_{z}=\frac{F_{\text{in},z}}{F_{\text{in},1}}=k_{\text{in},z}\cdot \frac{G_{\text{in},z}}{k_{\text{in},1}G_{\text{in},1}}\;\text{for}\;z=2,\ldots ,\mu_{i}, & \; \end{array}$$

where the *r*_*z*_ replace the kinetic parameters *k*_2_ to *k*_μ_*i*__. By means of Equations (8) and (9), we obtain

$$\begin{array}{ll} \underset{l}{\sum }F_{\text{out},l}=F_{\text{in},1}\cdot \left(1+\overset{\mu_{i}}{\underset{j=2}{\sum }}r_{\text{in},j}\right) & \; \end{array}$$

and therefore

(10) $$\begin{array}{ll} k_{\text{in},1}=\frac{1}{G_{\text{in},1}}\underset{l}{\sum }F_{\text{out},l}\cdot \frac{1}{1+\overset{\mu_{i}}{\underset{j=2}{\sum }}r_{j}}. & \; \end{array}$$

Since *G*_in, 1_ and *F*_out, l_ are positive and Equation (10) is a sum of positive contributions, a non-negative steady-state expression for *k*_in, 1_ is guaranteed. By means of Equation (9), the remaining flux parameters have to be substituted by the non-negative expressions

(11) $$\begin{array}{ll} k_{\text{in},z}=\frac{1}{G_{\text{in},z}}\underset{l}{\sum }F_{\text{out},l}\cdot \frac{r_{z}}{1+\overset{\mu_{i}}{\underset{j=2}{\sum }}r_{j}}\;\text{for}\;z=2,\ldots ,\mu_{i}. & \; \end{array}$$

For an outflux parameter, we analogously obtain

(12) $$\begin{array}{lll} k_{\text{out},1} & =\frac{1}{G_{\text{out},1}}\underset{j}{\sum }F_{\text{in},j}\cdot \frac{1}{1+\overset{\nu_{i}}{\underset{l=1}{\sum }}r_{l}}\;\text{and}\; & \; \end{array}$$

(13) $$\begin{array}{lll} k_{\text{out},z} & =\frac{1}{G_{\text{out},z}}\underset{j}{\sum }F_{\text{in},j}\cdot \frac{r_{z}}{1+\overset{\nu_{i}}{\underset{l=1}{\sum }}r_{l}}\;\text{for}\;z=2,\ldots ,\nu_{i}.\; & \; \end{array}$$

### 2.4. Algorithm for steady-state determination

In the previous sections, we showed how simple steady-state expressions can be obtained (Section 2.2), while positivity is likewise guaranteed (Section 2.3). In order to split one cycle of the steady-state graph and solve for a flux parameter, a *pair* (*x*_*i*_, *k*_*j*_) of state and flux parameter has to be chosen, which is not unique. In the following, we suggest an algorithm based on a classification of such pairs.

According to Definitions 5, 6 and Proposition 3, we associate each pair with one of four different types:

$$(x_{i},k_{j})\equiv \{\begin{array}{l} \text{Type 0},\text{ if }x_{i}\text{ appears in a CQ}\\ \text{Type 1},\;n_{k_{j}}=1\text{ and }\dim(x_{i})=1\\ \text{Type 2},\;n_{k_{j}}=1\text{ and }\dim(x_{i})>1\\ \text{Type 3},\text{ else}. \end{array}$$

**Figure 1:**
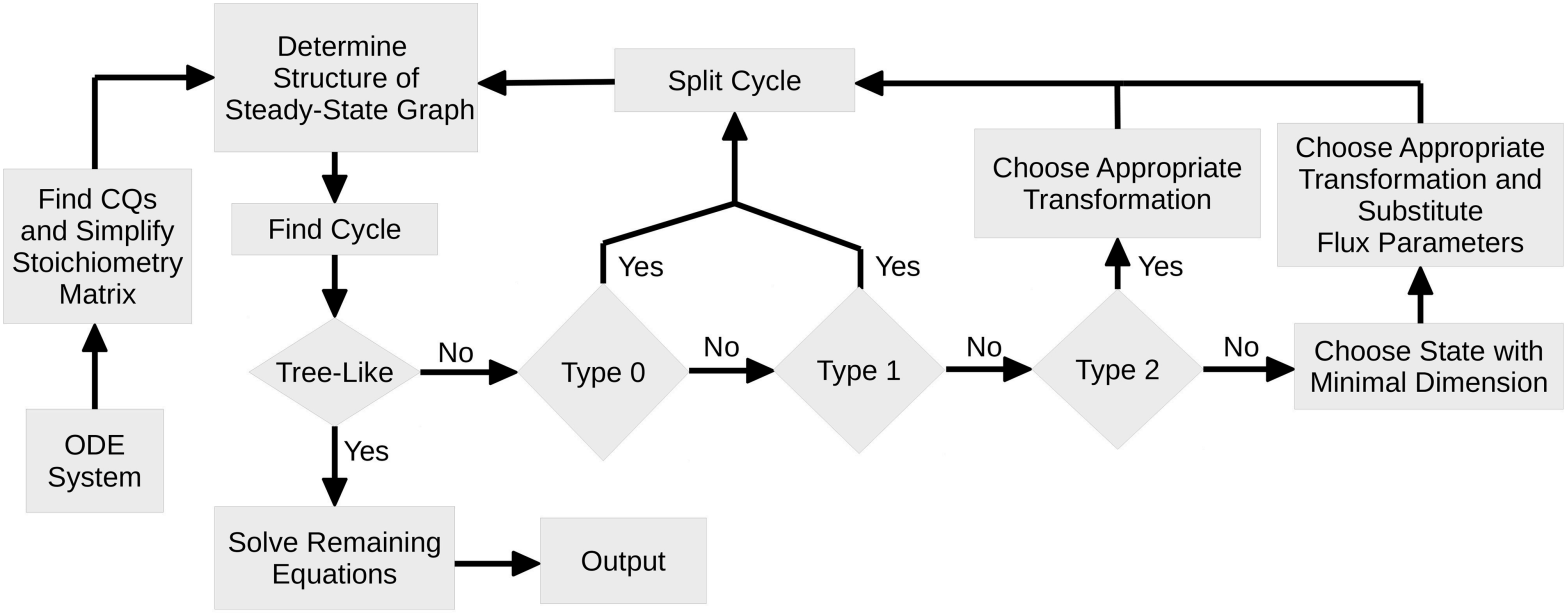
**Flowchart of Steady-State Determination: After identification of all CQs of the system, the algorithm performs a loop where in each pass one cycle of the steady-state graph is removed**. Since for each cycle the nodes are analyzed with regard to the number of in- and outflux rates, the graph structure as well as the structure of the steady-state equations is kept as simple as possible. Once the steady-state graph is tree-like, the remaining equations are solved and equations are returned.

Figure 1 shows a flowchart of the algorithm. At first, the set of CQs is computed for the ODE system serving as an input for the algorithm. If the graph is tree-like, the remaining equations are obtained according to *Proposition 2* and the complete set of steady-state equations is returned.

In the case of a pair of Type 0, the cycle can simply be removed by interpreting the corresponding state as a free variable. The CQ that is thereby used is removed from the set of CQs and cannot further contribute to the steady-state determination. Here, the flux parameters remain unaffected.

Unless the cycle cannot be directly split by means of a CQ, the corresponding steady-state equation, Equation (5), is solved for one of the μ_*i*_ + ν_*i*_ flux parameters by use of a pair of Type 1,2 or 3. In order to keep the steady-state solution as simple as possible, pairs of Type 1 are preferred, since this enables to split the cycle both without substituting the flux parameter by its steady-state expression, Equation (7), and without introducing flux ratios as new parameters, Equation (9).

If no pairs of Type 1 are available, the algorithm scans the steady-state graph for pairs of Type 2. In this case a parameter transformation is necessary in order to guarantee positivity of the solution. However, the flux parameter does not appear any more in the system and therefore has not to be substituted. In all three cases, Type 0, 1, or 2, the number of cycles of the steady-state graph is reduced.

If pairs of Type 2 are also not available, all pairs are of Type 3. In this case, it is not *a priori* clear which pair is the best choice. As a simply revisable choice, the algorithm then solves the steady-state equation of the state with minimal dimension. Subsequently, all further appearances of flux parameters have to be replaced by their particular transformation, Equations (11) or (13).

### 2.5. Calculating the conserved quantities and simplifying the stoichiometry matrix

In order to find CQs of the ODE system, linear combinations of rows of the stoichiometry matrix *S* can be analyzed. According to Equation (3), the *N*-dimensional ODE system can be written as

(14) $$\begin{array}{ll} ẋ=S\cdot F\left(x,p\right). & \; \end{array}$$

Multiplication of Equation (14) by an *N* × *N*-matrix *M* yields

(15) $$\begin{array}{ll} M\cdot ẋ=\tilde{S}\cdot F\left(x,p\right), & \; \end{array}$$

where the matrix $\tilde{S}=M\cdot S$ defines linear combinations of rows of *S*. For each row ${\tilde{S}}_{i}$ that is equal to zero, the quantity $M_{i}\cdot x=\underset{j}{\sum }M_{ij}x_{j}$ is conserved.

In fact, each set of linearly dependent rows of *S* implies a CQ. For some ODE systems, however, not all CQs can be derived from *S* without accounting for the flux vector *F*. Equation (14) can be written

(16) $$\begin{array}{ll} ẋ=C\left(p,x\right)\cdot x. & \; \end{array}$$

where *C*(*p, x*) is an *N* × *N*-matrix dependent on the parameters and states. Analyzing linear dependencies of *C*, all CQs of the form

(17) $$\begin{array}{ll} \underset{j}{\sum }a_{j}\left(x,p\right)\cdot x_{j}=\text{const.} & \; \end{array}$$

can be found, where the coefficients *a*_*j*_ might depend on states and parameters.

In order to determine symbolic expressions for the *a*_*j*_, we transpose the matrix *C* and numerically search for linearly dependent columns. All parameters and states appearing in *C*^*T*^ are replaced by random values to obtain a numeric matrix $C_{\text{ran}}^{T}$ for which a *QR*-decomposition is performed. The matrix *R* constitutes an upper triangular matrix, where the number of non-empty rows corresponds to the rank of *C*_ran_. The first column *R*_ℓ_ with *R*_ℓℓ_ = 0 is a linear combination of the columns *R*_*j* < ℓ_. Therefore, also the column *C*_ℓ_ is a linear combination of the columns *C*_*j* < ℓ_ implying that the equation system $\underset{j}{\sum }a_{j}C_{j}=0$ has a solution for the *a*_*j*_ with *a*_*j* > ℓ_ = 0 which can be calculated symbolically. Thus, the quantity *a*^*T*^ · *x* is conserved. Once a CQ has been found, one of the corresponding linearly dependent rows of the stoichiometry matrix is removed and the procedure is repeated. In most ODE systems, all CQs of the system can be obtained in that way. For all other cases, our Python code provides a possibility to manually specify CQs.

The idea of taking linear combinations of the stoichiometry matrix *S*, see Equations (14) and (15), can be augmented to simplify *S* for the calculation of steady-state expressions. For each matrix *M*, the original steady-state constraint, *S*·*F* = 0, is replaced by a new set of steady-state equations, $\tilde{S}\cdot F=0$. With a clever choice of *M*, these new steady-state equations might be structurally simpler than the original ones. With respect to our proposed algorithm, the matrix *M* should (1) minimize the overall number of entries in the new stoichiometry matrix $\tilde{S}$ and (2) prevent the creation of new cycles. In practice, the idea of linearly combining rows of the stoichiometry matrix can lead to structurally simpler steady-state expressions as we show by means of a small example in the Supplementary Material.

### 2.6. Numerically computed steady-states

Besides calculating steady-states analytically, roots of the steady-state constraint, Equation (2), can be computed numerically during each step of the optimization. Here, we perform Newton's method which is fast compared to the time of the ODE integration. The gradient information that is necessary within our deterministic optimization scheme is determined by the implicit function theorem, i.e., given the steady-state constraint

$$\begin{array}{ll} f\left(x_{0}\left(p\right),p\right)=0, & \; \end{array}$$

we derive the equation with respect to *p* and obtain

$$\begin{array}{ll} 0=\frac{\partial f}{\partial x_{0}}\cdot \frac{\partial x_{0}}{\partial p}+\frac{\partial f}{\partial p}\;\Rightarrow \;\frac{\partial x_{0}}{\partial p}=-{\left(\frac{\partial f}{\partial x_{0}}\right)}^{-1}\frac{\partial f}{\partial p}. & \; \end{array}$$

### 2.7. Technical remarks

The steady-state algorithm was implemented in Python by use of the libraries numpy and sympy. It can either be downloaded from the author's homepage as a Python code or can be used from within the R-packages dMod/cOde available from https://github.com/dkaschek/. Simulation of data and parameter estimation with analytical and numerical steady-states were performed in dmod.

## 3. Results

When calculating steady-state expressions for parameter estimation of ODE systems, several aspects have to be considered simultaneously. Most importantly, the parameter space is to be reduced as far as possible. Therefore, all available steady-state constraints should be taken into account. Since solving for state variables often leads to higher-order equations for which solutions are difficult to obtain, one has at least partially to solve for kinetic parameters. In doing so, the steady-state expressions often lead to negative parameter values for certain model specifications.

Due to mass balance, outfluxes contribute with a minus sign to the time derivative of the corresponding state. Provided that outflux rates are proportional to positive powers of their states, they contribute damping terms to the time-evolution of the state. However, if for a certain model specification the corresponding flux parameter is negative, the sign of the outflux term becomes positive which leads to an exploding model trajectory for the state.

In Section 3.1, we show how our steady-state approach determines simple steady-state equations for systems that lead to higher-order equations when solved for the state variables. In Section 3.2, we show how steady-state expressions with negative realizations lead to optimization problems and a significantly lower success rate, i.e., the probability to converge to a local or the global optimum. Non-linear ODE systems often have several steady-state solutions, when the steady-state equations are solved for the state variables. For parameter estimation, multiple steady-states constitute a problem, since all possible realizations have in principle to be followed up. By solving for kinetic parameters, our steady-state approach likewise avoids multiple solutions and improves the optimization as we show in Section 3.3.

### 3.1. Determination of non-negative steady-state expressions

To show the applicability of the presented steady-state approach, we investigate a toy model with six state variables and nine reactions of the form

$$\begin{array}{l} \begin{array}{ccc} \emptyset \overset{k_{0}}{\underset{}{\to }}A\; & A\overset{k_{1}}{\underset{}{\to }}B\; & B\overset{k_{2}}{\underset{}{\to }}A\\ A+A\overset{k_{3}}{\underset{}{\to }}C\; & C\overset{k_{4}}{\underset{}{\to }}\emptyset \; & B+C\overset{k_{5}}{\underset{}{\to }}D\\ D+G\overset{k_{6}}{\underset{}{\to }}F\; & B\overset{k_{7}\cdot F}{\underset{}{\to }}\emptyset \; & F\overset{k_{8}}{\underset{}{\to }}G.\\ \; \end{array} \end{array}$$

All reactions satisfy the law of mass action, the degradation of *B* is mediated by *F*. With these assumptions, one obtains the following ODE system

$$\begin{array}{lll} Ȧ & =k_{0}+k_{2}B-k_{1}A-k_{3}A^{2}\; & \;\\ Ḃ & =k_{1}A-k_{2}B-k_{5}BC-k_{7}BF\; & \;\\ Ċ & =k_{3}A^{2}-k_{5}BC-k_{4}C\; & \;\\ Ḋ & =k_{5}BC-k_{6}DG\; & \;\\ Ḟ & =k_{6}DG-k_{8}F\; & \;\\ Ġ & =k_{8}F-k_{6}DG.\; & \; \end{array}$$

The system contains one conserved quantity *F* + *G* = *const*, reflecting that the steady-state equations of *F* and *G* are not independent from each other. Therefore, the number of variables that have to be fixed by the steady-state is five. In order to obtain the corresponding steady-state equations, all time-derivatives of the states are set to zero. Although the single equations of this system are of degree two or lower, solving for the states leads to a sixth order polynomial equation, see Supplementary Material, for which no closed-form solution is available.

Table 4 summarizes how our steady-state solver determines a non-negative steady-state solution by partially solving for flux parameters. During the first loop, the cycle [*A, A*] of state *A* to itself is split. The pairs of *A* and its contributing flux parameters are all of Type 3, since there are two influx- and two outflux parameters of which at least one is appearing in the other steady-state equations, e.g., in the equation of *B*. The equation of *A* is solved for the influx parameter *k*_0_, whereby *k*_2_ is transformed and replaced by the new free parameter *r*_1_ = *k*_2_*B*∕*k*_0_, see first loop in Table 4. The appearance of *k*_2_ in the equation of *B* is substituted, whereas *k*_0_ has no further appearances.

**Table 4:** **Steps of steady-state determination for a model with six states and eight reactions**.

**Loop**	**Steady-state graph**	**Steady-state equations**	**Simplification**
1	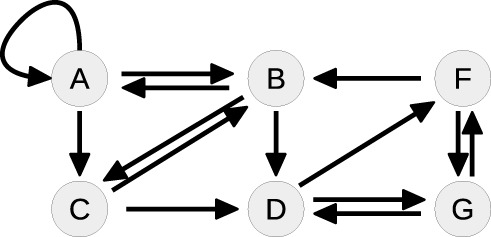	A: $0=k_{0}+k_{2}B-k_{1}A-k_{3}A^{2}$	Cycle: [*A*, *A*]
		B: 0 = *k*_1_*A* − *k*_2_*B* − *k*_5_*BC* − *k*_7_*BF*	Type 3
		C: $0=k_{3}A^{2}-k_{5}BC-k_{4}C$	$k_{0}=\frac{A^{2}k_{3}+Ak_{1}}{r_{1}+1}$
		D: 0 = *k*_5_*BC* − *k*_6_*DG*	
		F: 0 = *k*_6_*DG* − *k*_8_*F*	$k_{2}=r_{1}\frac{k_{0}}{B}=r_{1}\frac{A^{2}k_{3}+Ak_{1}}{B\left(r_{1}+1\right)}$
		G: 0 = *k*_8_*F* − *k*_6_*DG*	
2	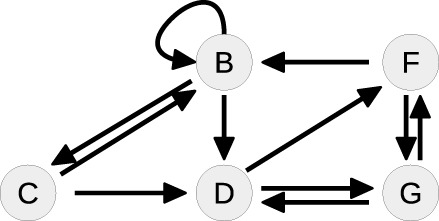	B: $0=k_{1}A\frac{1}{r_{1}+1}-k_{3}A^{2}\frac{r_{1}}{r_{1}+1}$	Cycle: [*B*, *B*]
		− *k*_5_*BC* − *k*_7_*BF*	Type 1
		C: $0=k_{3}A^{2}-k_{5}BC-k_{4}C$	
		D: 0 = *k*_5_*BC* − *k*_6_*DG*	$k_{1}=\frac{r_{1}+1}{A}k_{3}A^{2}\frac{r_{1}}{r_{1}+1}$
		F: 0 = *k*_6_*DG* − *k*_8_*F*	
		G: 0 = *k*_8_*F* − *k*_6_*DG*	+*k*_5_*BC* + *k*_7_*BF*]
3	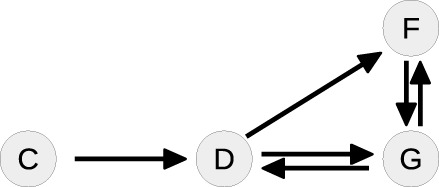	C: $0=k_{3}A^{2}-k_{5}BC-k_{4}C$	Cycle: [*D*, *G*, *D*]
		D: 0 = *k*_5_*BC* − *k*_6_*DG*	Type 0
		F: 0 = *k*_6_*DG* − *k*_8_*F*	*G* is part of CQ
		G: 0 = *k*_8_*F* − *k*_6_*DG*	
4	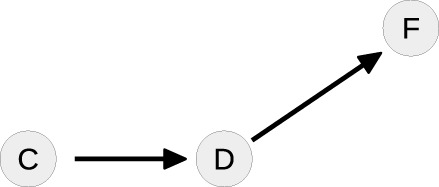	C: $0=k_{3}A^{2}-k_{5}BC-k_{4}C$	Tree-like
		D: 0 = *k*_5_*BC* − *k*_6_*DG*	
		F: 0 = *k*_6_*DG* − *k*_8_*F*	Solve for *C*, *D* and *F*

Whereas the state *A* is removed from the steady-state graph, the state *B* has become a new head of itself in consequence of the substitution. In the second loop, this new cycle [*B, B*] is split by solving the equation of *B* for the flux parameter *k*_1_. Here, the pair (*B, k*_1_) is of Type 1, since *k*_1_ is the only influx parameter and not appearing in the remaining equations.

In the next loop, the algorithm splits the cycle [*D, G, D*] by taking *G* as a free parameter, since it is part of the conserved quantity *F* + *G*. The remaining steady-state graph in the last loop is tree-like and therefore the steady-state equations can be derived according to *Proposition 2* starting with *C* which in this case serves as the root of the graph.

For simplification of writing, our steady-state solver outputs the equations in a specific order where fixed states or parameters may still appear in the equations below. In order to obtain a complete independent set of equations, one has to replace step by step. For the presented example, the ultimately obtained expressions are

$$\begin{array}{lll} F & =A^{2}\frac{k_{3}}{k_{8}}\frac{Bk_{5}}{Bk_{5}+k_{4}}\; & \;\\ D & =A^{2}\frac{k_{3}}{Gk_{6}}\frac{Bk_{5}}{Bk_{5}+k_{4}}\; & \;\\ C & =A^{2}\frac{k_{3}}{Bk_{5}+k_{4}}\; & \;\\ k_{1} & =Ak_{3}r_{1}+\frac{r_{1}+1}{A}\left(BCk_{5}+BFk_{7}\right)\; & \;\\ k_{2} & =r_{1}\frac{k_{3}A^{2}+k_{1}A}{B\left(r_{1}+1\right)}\; & \;\\ k_{0} & =\frac{k_{3}A^{2}+k_{1}A}{r_{1}+1},\; & \; \end{array}$$

where six parameters are fixed, while one additional parameter *r*_1_ can be chosen freely.

### 3.2. Minus signs imply a low convergence rate

For a given data set and a given ODE model, each parameter set determines the time-evolution of the states and its likelihood *L* can be computed based on the data. Here, parameter values are estimated by minimizing the negative log-likelihood function −log*L*. For the case of non-linear ODE models, several local optima may exist. In order to find the global optimum, we perform multi-start optimization in combination with a trust-region optimizer. A powerful optimization approach should have a high probability to find a local or the global optimum.

Let us consider an ODE system with four state variables and six reactions of the form

$$\begin{array}{lllllllllll} \emptyset & \overset{k_{0}}{\underset{}{\to }}A\; & \;A & \overset{k_{7}}{\underset{}{\to }}\emptyset \; & \;A+B & \overset{k_{2}}{\underset{}{\to }}C\; & \; & \; & \; & \; & \;\\ \emptyset & \overset{k_{1}}{\underset{}{\to }}B\; & \;C & \overset{k_{3}}{\underset{}{\to }}D\; & \;D & \overset{k_{4}}{\underset{}{\to }}\emptyset .\; & \; & \; & \; & \; & \; \end{array}$$

The corresponding ODE system is given by

$$\begin{array}{lll} Ȧ & =k_{0}-k_{7}A-k_{2}AB\; & \;\\ Ḃ & =k_{1}-k_{2}AB\; & \;\\ Ċ & =k_{2}AB-k_{3}C\; & \;\\ Ḋ & =k_{3}C-k_{4}D.\; & \; \end{array}$$

In order to test if negative steady-state expressions lead to optimization problems, we implemented four different steady-state parameterizations, see Table 5, and compared the success rate of parameter optimization. For each approach, six parameters are optimized as shown in Table 5. Besides the standard approach, i.e., exclusively solving for initial values, two other parameterizations were derived by solving the equation of state *B* for two different kinetic parameters, namely *k*_7_ and *k*_0_. The latter guarantees a non-negative steady-state solution. Apart from that, a fourth parameterization was constructed by adding the equation *k*_0_ = *k*_1_ + Δ_*k*_0__ to the standard steady-state formulation. In doing so, *k*_0_ is transformed such that *k*_0_ > *k*_1_, with the new free parameter Δ_*k*_0__ describing the difference between *k*_0_ and *k*_1_. This approach likewise implies positivity.

**Table 5:** **Several steady-state representations for a model with four states and six flux parameters**.

**Steady-state method**	**Steady-state equations**	**Parameters to be estimated**
Solved for initial values	*A* = *k*_0_ ∕ (*k*_2_*B* + *k*_7_)	*k*_1_, *k*_2_, *k*_3_, *k*_4_
	*B* = *k*_1_*k*_7_ ∕ (*k*_2_(*k*_0_ − *k*_1_))	
(Standard)	*C* = *k*_1_ ∕ *k*_3_	*k*_0_, *k*_7_
	*D* = *k*_1_ ∕ *k*_4_	
Equation of *B* solved for *k*_7_	*A* = *k*_0_ ∕ (*k*_2_*B* + *k*_7_)	*k*_1_, *k*_2_, *k*_3_, *k*_4_
	*k*_7_ = *Bk*_2_(*k*_0_ − *k*_1_)∕*k*_1_	
	*C* = *k*_1_ ∕ *k*_3_	*k*_0_, *B*
	*D* = *k*_1_ ∕ *k*_4_	
Equation of *B* solved for *k*_0_	*A* = *k*_0_ ∕ (*k*_2_*B* + *k*_7_)	*k*_1_, *k*_2_, *k*_3_, *k*_4_
	*k*_0_ = *k*_1_*k*_7_ ∕ (*Bk*_2_) + *k*_1_	
	*C* = *k*_1_ ∕ *k*_3_	*B*, *k*_7_
	*D* = *k*_1_ ∕ *k*_4_	
Standard	*k*_0_ = *k*_1_ + Δ_*k*_0__	*k*_1_, *k*_2_, *k*_3_, *k*_4_
	*A* = *k*_0_ ∕ (*k*_2_*B* + *k*_7_)	
with additional	*B* = *k*_1_*k*_7_ ∕ (*k*_2_(*k*_0_ − *k*_1_))	
parameter transformation	*C* = *k*_1_ ∕ *k*_3_	Δ_*k*_0__, *k*_7_
	*D* = *k*_1_ ∕ *k*_4_	

**Figure 2:**
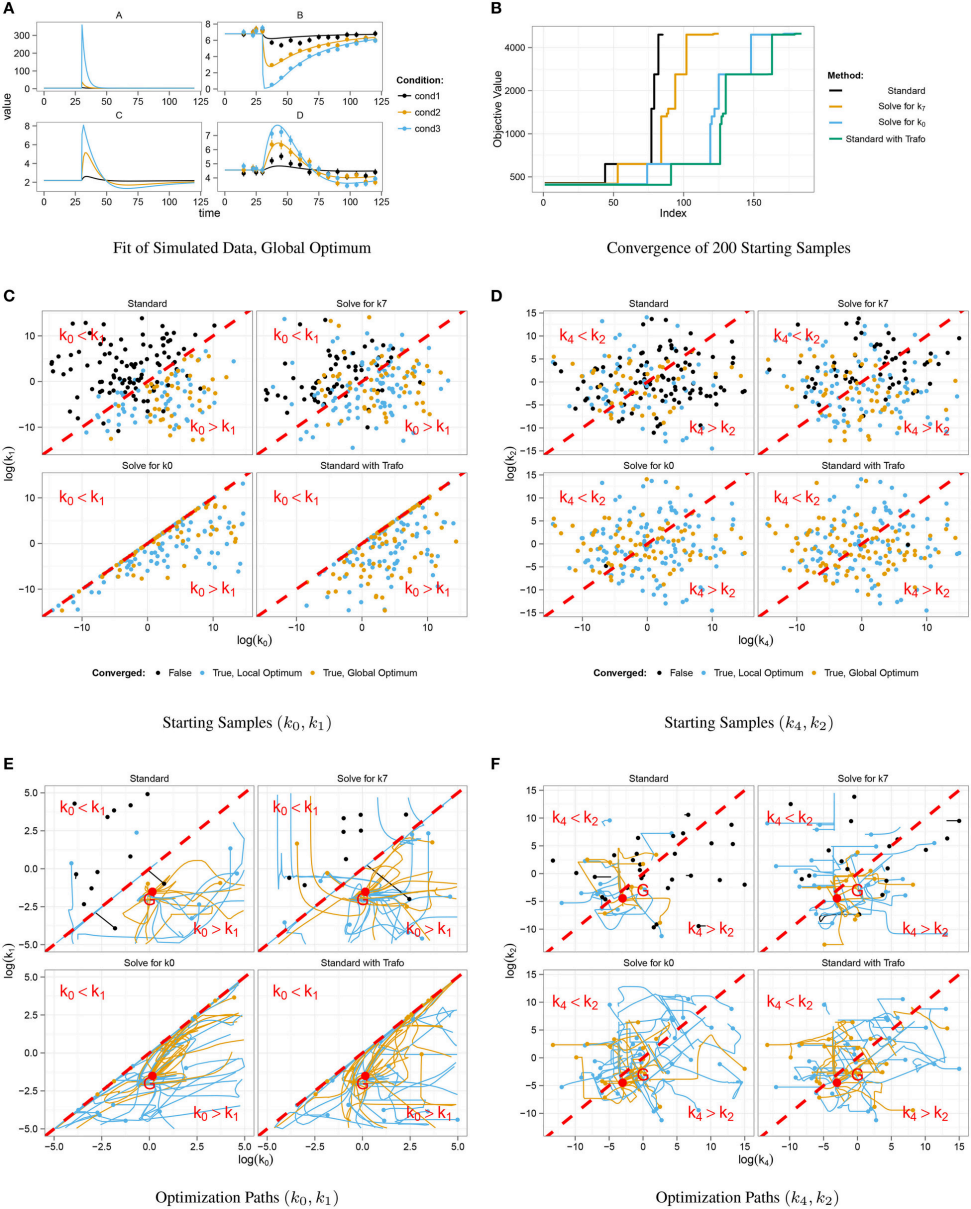
**Optimization results for different steady-state parameterizations**. Data was simulated for three different displacements of *A* at *t* = 30 **(A)**. Convergent fits for all four steady-state implementations were sorted by increasing objective value **(B)**. Steps correspond to local minima. Positive steady-state parameterizations show a considerably better convergence behavior. Starting samples are shown in different colors in **(C,D)**, indicating whether the corresponding optimization converged. Parameter paths starting with *k*_0_ < *k*_1_ did mostly not converge as opposed to samples with *k*_0_ > *k*_1_ which mostly converged to a local or the global optimum *G* **(E,F)**.

For simulation of data, we chose a set of kinetic parameters for ODE integration, initialized the system with its steady-state and excited it by displacement of *A* at time point *t* = 30. Data points were generated for 16 different time points by adding normally distributed noise to the model trajectories. In order to study a scenario with different experimental conditions, i.e., different stimulations, simulation was done for three different displacement values, compare *cond1, cond2*, and *cond3* in Figure 2A.

Figure 2A shows data points and trajectories of a model fit that reached the global optimum. For each steady-state parameterization, we optimized 200 different parameter samples and counted how often several optima were reached. In Figure 2B, all converged fits are shown in order of the objective value, in our case the negative log-likelihood value. Several steps corresponding to local optima appear for all steady-state parameterizations, the deepest step corresponds to the global optimum. It can be concluded that the two parameterizations without minus signs, i.e., *Solving for k*_0_ and *Standard with Trafo*, show a significantly better convergence than the other two. For example, in the parameterization with transformation, the global optimum was twice as often reached than in the standard approach.

In order to explain the convergence behavior of the different steady-state implementations, we analyzed the correlation between initial parameter guess and the success of optimization. The steady-state of the presented model is negative, if and only if *k*_0_ < *k*_1_. Figure 2C shows the starting samples along the parameter axes of *k*_0_ and *k*_1_ for all four steady-state parameterizations, colors indicate whether a sample did not converge (black) or did converge to a local (blue) or the global optimum (yellow). For comparison, Figure 2D shows starting samples along axes of parameters that do not affect the sign of the steady-state, namely *k*_2_ and *k*_4_. The sample distribution shows that samples with *k*_0_ > *k*_1_ have a high probability to converge, while samples with *k*_0_ < *k*_1_ tend to abort. On the other hand, the relation of *k*_2_ and *k*_4_ does not have a significant impact on the convergence probability. Furthermore, Figure 2C shows that the reparameterized steady-states prohibit sampling in the region with *k*_0_ < *k*_1_.

In addition to the starting samples, we analyzed the parameter paths during the optimization. Figures 2E,F show the paths for the first 50 starting samples with respect to the above used parameter axes. Parameter samples with *k*_0_ < *k*_1_ usually abort without any considerable steps in parameter space even though several samples cross the border *k*_0_ = *k*_1_ and proceed. In the opposite direction, some samples reach the border when started in the area with *k*_0_ > *k*_1_ and abort exactly at the border. The very most samples drawn with *k*_0_ > *k*_1_ converged to a local or the global optimum *G*. Again, Figure 2F underlines that the convergence behavior is unaffected by the relation of *k*_2_ and *k*_4_.

We conclude that steady-state parameterizations that lead to negative parameter values for certain model specifications constitute a severe issue for optimization. Due to the formulation of our steady-state algorithm, negative solutions are automatically avoided in the obtained steady-state expressions.

### 3.3. Dealing with multiplicity of steady-states

Let us consider a system with three state variables and seven reactions of the form

$$\begin{array}{llllllllllllll} \emptyset & \overset{k_{0}}{\underset{}{\to }}A\; & \;A & \overset{k_{1}}{\underset{}{\to }}\emptyset \; & \;\emptyset & \overset{k_{2}\cdot A}{\underset{}{\to }}B\; & \;\emptyset & \overset{k_{3}\cdot C}{\underset{}{\to }}A\; & \; & \; & \; & \; & \; & \;\\ B & \overset{k_{4}}{\underset{}{\to }}\emptyset \; & \;\emptyset & \overset{k_{5}\cdot A\cdot B}{\underset{}{\to }}C\; & \;C & \overset{k_{6}}{\underset{}{\to }}\emptyset .\; & \; & \; & \; & \; & \; & \; \end{array}$$

The production of *B* is mediated by *A*, production of *A* is mediated by *C* and production of *C* is mediated by both *A* and *B*. The corresponding ODE system is given by

(18) $$\begin{array}{lll} Ȧ & =k_{0}+k_{3}C-k_{1}A\; & \;\\ Ḃ & =k_{2}A-k_{4}B\; & \; \end{array}$$

$$\begin{array}{lll} Ċ & =k_{5}AB-k_{6}C.\; & \; \end{array}$$

For this system, the steady-state can still be analytically solved for the states *A*, *B* and *C*. The solution reads

(19) $$\begin{array}{lll} C_{1∕2} & =\frac{1}{2k_{2}k_{3}^{2}k_{5}}\left(\frac{1}{k_{1}^{2}k_{4}k_{6}}\Delta +2k_{0}k_{2}k_{3}k_{5}\pm \sqrt{\Delta }\right)\; & \;\\ B_{1∕2} & =\frac{1}{2k_{1}k_{3}k_{4}k_{5}}\left(k_{1}^{2}k_{4}k_{6}\pm \sqrt{\Delta }\right)\; & \; \end{array}$$

$$\begin{array}{lll} A_{1∕2} & =\frac{1}{2k_{1}k_{2}k_{3}k_{5}}\left(k_{1}^{2}k_{4}k_{6}\pm \sqrt{\Delta }\right),\; & \; \end{array}$$

with the discriminant $\Delta =k_{1}^{2}k_{4}k_{6}\cdot \left(k_{1}^{2}k_{4}k_{6}-4k_{0}k_{2}k_{3}k_{5}\right)$. For Δ > 0, two positive steady-state solutions *S*_1_ = (*A*_1_, *B*_1_, *C*_1_) and *S*_2_ = (*A*_2_, *B*_2_, *C*_2_) are obtained, while the system has no real steady-state for Δ < 0.

Linear stability analysis reveals that solution *S*_1_ is unstable and solution *S*_2_ is stable, see Supplementary Material. If several steady-state solutions exist, only one of them can be chosen for an optimization run at a time. Here, the stable solution was chosen.

For this system, the issue of multiplicity can easily be solved, however, for more complicated systems several stable solutions might exist. Stable solutions might even switch to unstable solutions along the optimization path, e.g., in case of a Hopf bifurcation. For higher-order equations, analytical solutions become unfeasible and numerical steady-state computation comes into play. However, since the numerical root finding is performed by means of Newton's method, the result depends on initial guesses for *A*, *B* and *C*. Consequently, it is not clear which of the solutions is obtained and stability of the retrieved steady-state is not guaranteed. As we will show, coexistence of stable and unstable steady-state solutions leads to a reduced convergence probability in the numerical approach.

Unlike solving the steady-state equations for *A*, *B* and *C*, the steady-state expressions obtained by our proposed approach are

(20) $$\begin{array}{llllllll} C=A^{2}\frac{k_{2}k_{5}}{k_{4}k_{6}} & \; & B=A\frac{k_{2}}{k_{4}} & \; & k_{1}=A\frac{k_{2}k_{3}k_{5}}{k_{4}k_{6}}+\frac{k_{0}}{A}, & \; & \; & \; \end{array}$$

where the kinetic parameter *k*_1_ is fixed, while the initial value of *A* is taken as a free parameter. The obtained solution is unique, since the steady-state equations are linear in the parameters *B*, *C* and *k*_1_.

In general, our approach avoids multiple-steady-states by choosing a combination of parameters for which the steady-state equations are linear. In doing so, no solution is neglected as long as all steady-state equations are fulfilled. As an analogon, let us consider a single algebraic equation of the form *ab*^3^ + *cb*^2^ + *db* + *f* = 0, with the five parameters *a*, *b*, *c*, *d* and *f*. On the one hand this equation can be solved for *b* whereby multiple solutions are obtained. On the other hand it can be solved for one of the parameters *a*, *c*, *d* or *f* for which the equation is linear leading to a unique solution.

In the following, we compare the convergence behavior of three different steady-state implementations, namely *Standard*, i.e., analytically solved for the states, *Numeric*, i.e., numerically solved for the states, and *Proposed*, i.e., our steady-state approach with positive solutions. For the former two implementations, the seven kinetic parameters *k*_0_ to *k*_6_ are estimated, whereas for the proposed approach, the initial value of *A* in estimated instead of *k*_1_, compare Equation (20).

Since natural systems are always subject to external noise, unstable steady-states are never realized by the system. Therefore, data was simulated by means of the stable steady-state solution. Analogously to Section 3.2, three different displacements of the state *A* were triggered at time point *t* = 30 to excite the system. Here, data points were generated for eight different time points.

**Figure 3:**
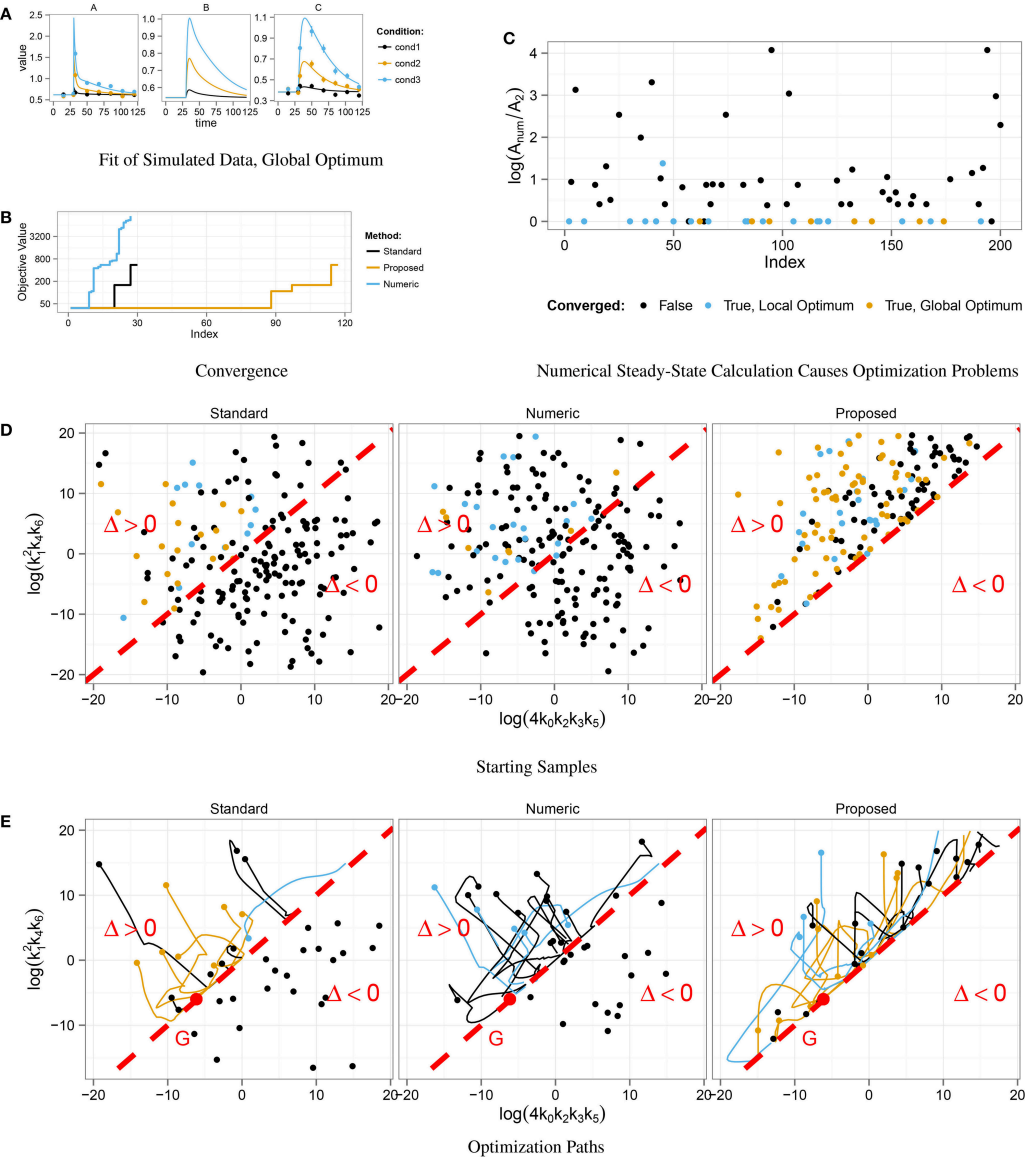
**Optimization in the context of multiple steady-states**. Data was simulated for three different displacements of *A* at *t* = 30 **(A)**. Convergent fits from 200 starting samples for three different steady-state implementations were sorted by their final objective value **(B)**. Fits that did not converge are not shown. In about 10% of the fits, the *Standard* and the *Numeric* approach converged, in the *Proposed* approach nearly 50% did. For each end point of the 200 numerical optimizations, the ratio *A*_*num*_ ∕ *A*_2_ between the numerical solution *A*_*num*_ and the stable, analytical steady-state solution *A*_2_ was computed **(C)**. For *A*_*num*_ ∕ *A*_2_ > 0, the numerical root calculation converged to the unstable steady-state which effects the abort of the optimization. Starting samples are shown in different colors, indicating whether the corresponding optimization converged. Many parameter samples starting with discriminant Δ < 0 did not converge, while most of the samples with Δ > 0 converged to the global optimum *G*, **(D,E)**.

In order to test the convergence behavior, we started 200 fits from randomly chosen parameter samples. Figure 3A shows an example fit that converged to the global optimum. The optimization result of the three steady-state approaches is compared in Figure 3B. Steps correspond to local optima. In our approach, nearly half of the samples converged to the global optimum, whereas only about 10% of the fits converged in the standard approach and even less in the numeric approach.

Similar to Section 3.2, we analyzed the correlation between initial parameter guess and success in optimization. Figure 3D shows the distribution of starting samples with respect to the sign of the discriminant Δ. For Δ < 0 the discriminant of the standard steady-state expression, Equation (19), becomes negative, and all starting samples drawn from this region did not converge. In addition, Figure 3E shows that the optimization of these samples directly aborted, since the path did not take any or at most a very small step in parameter space.

Furthermore, we analyzed the correlation between the coexistence of stable and unstable steady-state solutions and the success of the numerical approach. During each optimization step, the root of the ODE's right-hand side is computed for the current parameter values. Depending on the initial guess, either the stable or the unstable solution is obtained. In order to see, if the unstable solution causes optimization aborts, we chose state *A* as a representative and compared numerically and analytically calculated values at the end of the optimization path. The numerically calculated value *A*_*num*_ was taken from the root calculation by Newton's method and the value *A*_2_ of the stable steady-state was calculated by means of Equation (19) with the corresponding parameter values. Figure 3C shows ratios *A*_*num*_ ∕ *A*_2_ for all fits. If *A*_*num*_ ∕ *A*_2_ = 0, the stable steady-state was obtained, while *A*_*num*_ ∕ *A*_2_ > 0 implies that the unstable solution was obtained. Since nearly all fits that reached the unstable solution did not converge, we conclude that the coexistence of a second unstable steady-state causes optimization aborts in the numerical approach of steady-state determination.

Both problems arising from the existence of multiple steady-states, i.e., negative discriminants and stable vs. unstable steady-states, are automatically circumvented by our steady-state algorithm resulting in a superior convergence rate during parameter estimation.

## 4. Discussion and conclusion

Parameter estimation in non-linear ODE models of biological systems has to deal with several local optima and a high-dimensional parameter space. In order to reduce the number of parameters, steady-state constraints are taken into account. Deterministic algorithms search for the global optimum by performing the optimization with multiple starting samples. The way of implementing steady-states, i.e., the exact parameterization, has an impact on the convergence probability of a randomly chosen starting sample. If optimizations tend to abort before reaching an optimum, many starting samples are necessary to find the best possible fit. Since incorporation of steady-state information shifts parameter distributions and contributes to gradient information, the exact steady-state parametrization plays a crucial role in optimization.

For many systems, steady-state equations lead to higher-order polynomial equations when being solved for the state variables. To exploit the full steady-state information, equations can be partially solved for kinetic parameters. If the obtained steady-state expressions yield negative values for certain parameter specifications, those might lead to rapidly growing solutions for the ODE system. We showed that negative parameter values have a considerable, negative impact on the success of the optimization.

In many applications, multiplicity and multi-stability of the steady-state constitutes the relevant question. In the case of parameter estimation, however, multiple steady-states complicate the estimation process. For the standard approach of solving steady-state equations for the state variables, all solutions principally have to be considered and optimization has to be performed for all possibilities. For the numerical implementation of steady-states also unstable steady-state solutions constitute a problem, since the numerical root finding method might converge to the unstable solution. In our case, the convergence probability dropped by 80%.

In this work, we presented an algorithm that derives steady-state expressions and circumvents negative and multiple solutions by construction. The approach covers the most common classes of ODE models consisting of e.g., mass-action kinetics, inhibition terms, Michaelis-Menten or Hill-type equations. By means of graph theory, cyclic dependencies between dynamical variables, e.g., positive or negative feedbacks inside a signaling cascade that lead to polynomial equations of order two or higher are removed by solving for kinetic parameters for which the equations are linear. In order to guarantee positivity of all solutions, the algorithm performs appropriate parameter transformations replacing kinetic parameters by ratios of participating fluxes. Our approach experiences a major limitation if simultaneously, the size of the ODE model becomes large and combinations of several in- and outflux parameters contribute to multiple states. Then, the algorithm is not able to find a strictly positive solution for the system. Furthermore, since the algorithm solves for rate parameters, it might not be applicable if solving for parameters is not allowed due to other reasons, e.g., if rate parameters must take certain fixed values.

In summary, our approach enables steady-state calculation for models with many cyclic dependencies that lead to higher-order polynomial equations when solved for state variables. Multiplicity and multi-stability are avoided and positivity of the solution is guaranteed. The parameter space is reduced by the number of independent steady-state equations while the nice convergence behavior is preserved.

## Author contributions

MR developed, implemented and tested the method. MR, JT, and DK together wrote the paper.

## Funding

This work was supported by the Federal Ministry of Education and Research (BMBF), by the MIP-DILI project, Innovative Medicines Initiative Joint Undertaking under grant agreement No. 115336-2 and by the ReelinSys project, Systems biology of Reelin-associated neuropsychiatric disorders, under No. 0316174D.

### Conflict of interest statement

The authors declare that the research was conducted in the absence of any commercial or financial relationships that could be construed as a potential conflict of interest.
