# Supplementary material for: Customized Steady-State Constraints for Parameter Estimation in Non-Linear Ordinary Differential Equation Models
Source: Front Cell Dev Biol. 2016 May 11;4:41. doi: 10.3389/fcell.2016.00041 (PMC4863410; doi:10.3389/fcell.2016.00041)
Supplement: Supplementary file 1 [file Presentation1.PDF]

1 SIMPLIFYING THE STOICHIOMETRY MATRIX

For a model with four state variables and seven reactions

$$\emptyset \xrightarrow{k_0} A \qquad A \xrightarrow{k_1} \emptyset \qquad \emptyset \xrightarrow{k_2} B \qquad B \xrightarrow{k_3} \emptyset$$
$$A + B \xrightarrow{k_4} C + D \qquad C \xrightarrow{k_5} B \qquad D \xrightarrow{k_6} A\,,$$

the ODE system has the form

$$\dot{x} = S \cdot F$$
$$\frac{d}{dt}\begin{pmatrix} A \\ B \\ C \\ D \end{pmatrix} = \begin{pmatrix} 1 & -1 & 0 & 0 & -1 & 0 & 1 \\ 0 & 0 & 1 & -1 & -1 & 1 & 0 \\ 0 & 0 & 0 & 0 & 1 & -1 & 0 \\ 0 & 0 & 0 & 0 & 1 & 0 & -1 \end{pmatrix} \cdot \begin{pmatrix} k_0 \\ k_1 A \\ k_2 \\ k_3 B \\ k_4 AB \\ k_5 C \\ k_6 D \end{pmatrix}\,. \tag{1}$$

Without simplifying the stoichiometry matrix, the proposed algorithm returns the steady-state expressions

$$B = \frac{k_2}{k_3} \qquad k_4 = k_1 r_A \frac{1}{B} \qquad k_5 = ABk_4 \frac{1}{C}$$
$$k_6 = (ABk_4 + Ak_1)\frac{r_A}{(r_A+1)D} \qquad k_0 = (ABk_4 + Ak_1)\frac{1}{r_A+1}\,.$$

In the following we choose a particular linear combination of rows of the stoichiometry matrix. Multiplying Eq. (1) by

$$M = \begin{pmatrix} 1 & 0 & 1 & 0 \\ 0 & 1 & 0 & 1 \\ 0 & 0 & 1 & 0 \\ 0 & 0 & 0 & 1 \end{pmatrix}$$

leads to the an ODE system

$$M \cdot \dot{x} = M \cdot S \cdot F$$
$$M \cdot \dot{x} = \tilde{S} \cdot F$$
$$\frac{d}{dt}\begin{pmatrix} A + D \\ B + C \\ C \\ D \end{pmatrix} = \begin{pmatrix} 1 & -1 & 0 & 0 & 0 & 0 & 0 \\ 0 & 0 & 1 & -1 & 0 & 0 & 0 \\ 0 & 0 & 0 & 0 & 1 & -1 & 0 \\ 0 & 0 & 0 & 0 & 1 & 0 & -1 \end{pmatrix} \cdot \begin{pmatrix} k_0 \\ k_1 A \\ k_2 \\ k_3 B \\ k_4 AB \\ k_5 C \\ k_6 D \end{pmatrix}$$

which has simplified stoichiometry matrix $\tilde{S}$. In this case, the steady-state expressions are

$$D = \frac{k_0 k_2 k_4}{k_1 k_3 k_6} \qquad C = \frac{k_0 k_2 k_4}{k_1 k_3 k_5}$$
$$B = \frac{k_2}{k_3} \qquad A = \frac{k_0}{k_1}\,.$$

2 OBTAINING 6TH-ORDER EQUATION

For a model with six state variables and nine reactions

$$\emptyset \xrightarrow{k_0} A \qquad A \xrightarrow{k_1} B \qquad B \xrightarrow{k_2} A \qquad A + A \xrightarrow{k_3} C \qquad C \xrightarrow{k_4} \emptyset$$
$$B + C \xrightarrow{k_5} D \qquad D + G \xrightarrow{k_6} F \qquad B \xrightarrow{k_7 \cdot F} \emptyset \qquad F \xrightarrow{k_8} G\,,$$

the steady-state equations hold

$$\begin{aligned}
0 &= k_0 + k_2 B - k_1 A - k_3 A^2 \\
0 &= k_1 A - k_2 B - k_5 BC - k_7 BF \\
0 &= k_3 A^2 - k_5 BC - k_4 C \\
0 &= k_5 BC - k_6 DG \\
0 &= k_6 DG - k_8 F \\
0 &= k_8 F - k_6 DG\,.
\end{aligned}$$

Choose G as a free parameter. Fix $F = \frac{k_6 DG}{k_8}$.

$$\begin{aligned}
0 &= k_0 + k_2 B - k_1 A - k_3 A^2 \\
0 &= k_1 A - k_2 B - k_5 BC - k_7 B \frac{k_6 DG}{k_8} \\
0 &= k_3 A^2 - k_5 BC - k_4 C \\
0 &= k_5 BC - k_6 DG
\end{aligned}$$

Fix $D = \frac{k_5 BC}{k_6 G}$.

$$\begin{aligned}
0 &= k_0 + k_2 B - k_1 A - k_3 A^2 \\
0 &= k_1 A - k_2 B - k_5 BC - k_7 B^2 C \frac{k_5}{k_8} \\
0 &= k_3 A^2 - k_5 BC - k_4 C
\end{aligned}$$

Fix $C = \frac{k_3 A^2}{k_5 B + k_4}$.

$$
\begin{aligned}
0 &= k_0 + k_2 B - k_1 A - k_3 A^2 \\
0 &= k_1 A - k_2 B - k_5 B \frac{k_3 A^2}{k_5 B + k_4} - k_7 B^2 \frac{k_3 A^2}{k_5 B + k_4} \frac{k_5}{k_8}
\end{aligned}
$$

Multiplying by $(k_5 B + k_4)$ yields

$$
\begin{aligned}
0 &= k_0 + k_2 B - k_1 A - k_3 A^2 \\
0 &= (k_5 B + k_4) k_1 A - (k_5 B + k_4) k_2 B - k_5 B k_3 A^2 - k_7 B^2 k_3 A^2 \frac{k_5}{k_8}
\end{aligned}
$$

Fixing $B = \frac{k_1 A + k_3 A^2 - k_0}{k_2}$, a 6-th order polynomial equation for A is obtained.

3 STABLE AND UNSTABLE SOLUTION

Let us consider the following steady-state constraint

$$
\begin{aligned}
0 &= k_0 + k_3 C - k_1 A \\
0 &= k_2 A - k_4 B \\
0 &= k_5 A B - k_6 C \,.
\end{aligned}
$$

Fixing $C = \frac{k_5 A B}{k_6}$ and $B = \frac{k_2 A}{k_4}$ yields

$$
0 = k_0 + k_3 \frac{k_5 k_2}{k_4 k_6} A^2 - k_1 A \,. \tag{2}
$$

Solving the quadratic equation for A leads to

$$
A_{\pm} = \frac{1}{2 k_1 k_2 k_3 k_5} \left(k_1^2 k_4 k_6 \pm \sqrt{\Delta} \right) \,,
$$

with $\Delta = k_1^2 k_4 k_6 \cdot \left(k_1^2 k_4 k_6 - 4 k_0 k_2 k_3 k_5 \right)$.

In order to perform linear stability analysis, we set

$$
A = \frac{1}{2 k_1 k_2 k_3 k_5} \left(k_1^2 k_4 k_6 \pm \sqrt{\Delta} \right) + \delta A \,,
$$

which together with Eq. (2) leads to

$$\begin{aligned}
\dot{A} &= k_0 + k_3\frac{k_5k_2}{k_4k_6}\left(\frac{1}{2k_1k_2k_3k_5}\left(k_1^2k_4k_6 \pm \sqrt{\Delta}\right) + \delta A\right)^2 - \frac{1}{2k_2k_3k_5}\left(k_1^2k_4k_6 \pm \sqrt{\Delta}\right) - k_1\delta A \\
&= k_0 + \frac{\left(k_1^2k_4k_6 \pm \sqrt{\Delta}\right)^2}{4k_1^2k_2k_3k_4k_5k_6} + \frac{\left(k_1^2k_4k_6 \pm \sqrt{\Delta}\right)}{k_1k_4k_6}\delta A - \frac{\left(k_1^2k_4k_6 \pm \sqrt{\Delta}\right)}{2k_2k_3k_5} - k_1\delta A \\
&= k_0 + \frac{\left(k_1^2k_4k_6 \pm \sqrt{\Delta}\right)^2 + 4k_1k_2k_3k_5\left(k_1^2k_4k_6 \pm \sqrt{\Delta}\right)\delta A - 2k_1^2k_4k_6\left(k_1^2k_4k_6 \pm \sqrt{\Delta}\right)}{4k_1^2k_2k_3k_4k_5k_6} - k_1\delta A \\
&= k_0 + \frac{\left(k_1^2k_4k_6 \pm \sqrt{\Delta}\right)^2 - 2k_1^2k_4k_6\left(k_1^2k_4k_6 \pm \sqrt{\Delta}\right)}{4k_1^2k_2k_3k_4k_5k_6} \pm \frac{\sqrt{\Delta}}{k_1k_4k_6}\delta A \\
&= k_0 + \frac{\left(k_1^2k_4k_6\right)^2 \pm 2k_1^2k_4k_6\sqrt{\Delta} + \Delta - 2k_1^2k_4k_6\left(k_1^2k_4k_6 \pm \sqrt{\Delta}\right)}{4k_1^2k_2k_3k_4k_5k_6} \pm \frac{\sqrt{\Delta}}{k_1k_4k_6}\delta A \\
&= k_0 + \frac{\Delta - \left(k_1^2k_4k_6\right)^2}{4k_1^2k_2k_3k_4k_5k_6} \pm \frac{\sqrt{\Delta}}{k_1k_4k_6}\delta A \\
&= \pm\frac{\sqrt{\Delta}}{k_1k_4k_6}\delta A
\end{aligned}$$

which signifies that independent of the parameterization, A_+ is instable and A_- is stable.
